# Pro-inflammatory macrophages coupled with glycolysis remodel adipose vasculature by producing platelet-derived growth factor-B in obesity

**DOI:** 10.1038/s41598-019-57368-w

**Published:** 2020-01-20

**Authors:** Yasuhiro Onogi, Tsutomu Wada, Akira Okekawa, Takatoshi Matsuzawa, Eri Watanabe, Keisuke Ikeda, Minoru Nakano, Munehiro Kitada, Daisuke Koya, Hiroshi Tsuneki, Toshiyasu Sasaoka

**Affiliations:** 1 Department of Clinical Pharmacology, University of Toyama, Sugitani 2630 Toyama, Japan; 2 Department of Biointerface Chemistry, University of Toyama, Sugitani 2630 Toyama, Japan; 3 Department of Diabetology and Endocrinology, Kanazawa Medical University, 1-1 Daigaku, Uchinada Ishikawa, 920-0293 Japan

**Keywords:** Metabolic disorders, Obesity

## Abstract

Adipose tissue macrophages (ATMs) play a central role in tissue remodeling and homeostasis. However, whether ATMs promote adipose angiogenesis in obesity remains unclear. We examined the impact of ATMs deletion on adipose angiogenesis and tissue expansion in the epididymal white adipose tissue (eWAT) of high-fat diet (HFD)-fed mice by using liposome-encapsulated clodronate. We further elucidated the induction mechanisms of platelet-derived growth factor (PDGF)-B in macrophages in response to obesity-associated metabolic stresses, since it plays a significant role in the regulation of pericyte behavior for the initiation of neoangiogenesis during tissue expansion. ATM depletion prevented adipose tissue expansion in HFD-fed mice by inhibiting pericyte detachment from vessels, resulting in less vasculature in eWAT. The lipopolysaccharide (LPS) stimulation and high glucose concentration augmented glucose incorporation and glycolytic capacity with the induction of *Pdgfb* mRNA. This effect was mediated through extracellular signal-regulated kinase (ERK) among mitogen-activated protein kinases coupled with glycolysis in RAW264.7 macrophages. The *Pdgfb* induction system was distinct from that of inflammatory cytokines mediated by mechanistic target of rapamycin complex 1 (mTORC1) and NFκB signaling. Thus, obesity-associated hyperglycemia and chronic inflammation fuels ERK signaling coupled with glycolysis in pro-inflammatory macrophages, which contribute to the expansion of eWAT through PDGF-B-dependent vascular remodeling.

## Introduction

The excessive expansion of white adipose tissue (WAT) disturbs systemic metabolic homeostasis. Insufficient vascular development in obese WAT induces chronic inflammation, resulting in systemic insulin resistance and metabolic dysfunction^1–6^. Hypoxia due to vascular rarefaction in growing WAT is one trigger inducing pro-angiogenic factors^7^. Vascular endothelial growth factor (VEGF)-A is a well-characterized pro-angiogenic factor that stimulates endothelial proliferation^1,2^. Endothelial proliferation in mature vessels is suppressed by pericytes and perivascular cells^7–10^. We recently reported an important role for platelet-derived growth factor (PDGF)-B in pericyte behavior during WAT expansion in obesity^11^. Increased PDGF-B stimulates the dissociation of CD13^+^ pericytes from adipose vessels via its receptor PDGFRβ, resulting in endothelial cell proliferation at pericyte-detached vessel areas in WAT during diet-induced obesity^11^.

F4/80^+^CD11c^+^ pro-inflammatory macrophages promote chronic inflammation by producing inflammatory cytokines, including tumor necrosis factor (TNF) α and interleukin (IL)-6^12,13^. Macrophages secrete angiogenic factors under tissue remodeling conditions in tumors, wounded skin, bone, and adipose tissues^5,14–16^. Consistent with these findings, we reported the up-regulated expression of PDGF-B in F4/80^+^CD11c^+^ macrophages in the eWAT of mice fed a high-fat diet (HFD) in a time-dependent manner^11^. Moreover, macrophages accumulated in stroma with few vessel-associated pericytes in obese WAT^11^. However, it remains unclear whether adipose tissue macrophages are involved in pericyte detachment, which promotes angiogenesis in WAT during the progression of obesity.

Increasing evidences show that cellular metabolism plays a key role in energy production and the control of cell function, particularly in macrophages^17–22^. Classically activated macrophages stimulated with lipopolysaccharide (LPS) (plus interferon γ) incorporate a large amount of glucose from aerobic glycolysis to meet energy demands. This change in cellular metabolism is attributed to a dysfunctional tricarboxylic acid cycle caused by the accumulation of citrate and succinate^18^. The promotion of glycolysis and its branched pathway, the pentose phosphate pathway, is necessary for cytokine production through the generation of reactive oxygen species (ROS) in classically activated macrophages^17,19–22^. Furthermore, human monocytes and murine macrophages produce greater amounts of cytokines under high glucose conditions^23,24^. However, the mechanisms by which PDGF-B is restrictedly produced by adipose tissue macrophages (ATMs) that exist in a cellular environment under metabolic stress imposed by abundant glucose and free fatty acids remain unclear.

We reported a crucial role for PDGF-B in the vascular remodeling of visceral adipose tissue in obesity^11^. However, the cells producing PDGF-B and its induction mechanisms in obese adipose tissue remain unknown. To further understand adipose tissue biology in obesity, we attempted to clarify these issues. In the present study, we demonstrated the principal role of ATMs in pericyte detachment, an initial event in adipose angiogenesis in obesity, using liposome-encapsulated clodronate, a macrophage-depletion reagent. We also clarified the divergence of signals inducing the expression of *Pdgfb* and pro-inflammatory cytokines in macrophages. Obesity-associated metabolic stress provoked metabolic reprogramming towards glycolysis in macrophages. Extracellular signal-regulated kinase (ERK) signaling coupled with glycolytic activity plays an important role in the induction of *Pdgfb* expression in macrophages, whereas mechanistic target of rapamycin complex 1 (mTORC1) and p65 NFκB signaling are mainly involved in inflammatory responses. Acidic conditions generated by lactic acid, a by-product of glycolysis, augmented LPS-induced *Pdgfb* expression without any changes in the expression of inflammatory cytokines. These results indicate that ATMs handle optimal signaling pathways coupled with glycolysis in response to environmental cues and contribute to the progression of chronic inflammation and vascular remodeling in obese adipose tissue.

## Results

### ATMs play significant role in adipose vascular remodeling and tissue expansion of diet-induced obese mice

**Figure 1:**
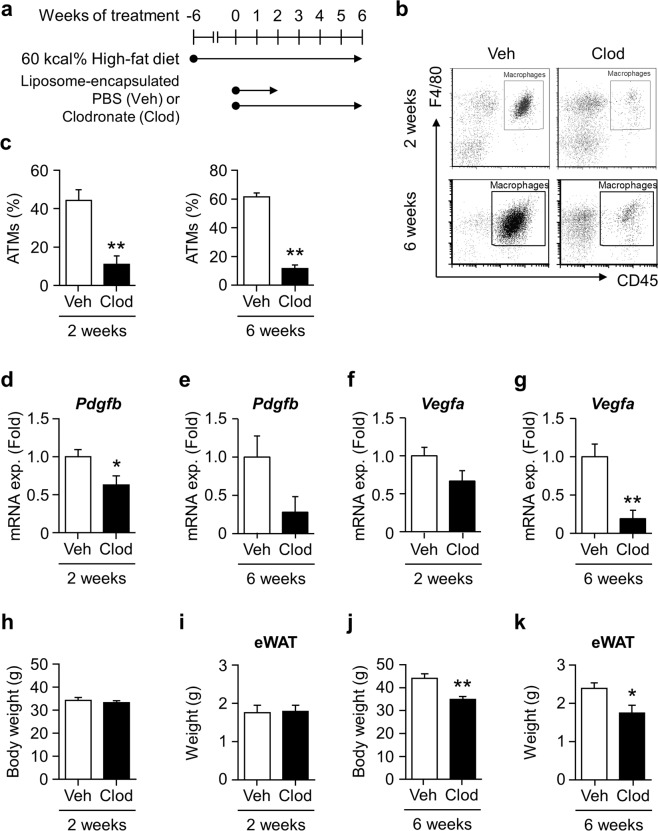
Impact of adipose macrophage deletion on gene expression and tissue weight in the WAT of HFD-fed mice. (**a**) Experimental protocol for the liposome injection. Liposome-encapsulated clodronate (Clod) or PBS (Veh) was administered twice a week for 2 or 6 weeks to mice fed HFD for 6 weeks, and maintained on HFD during the administration protocol. (**b**) Representative scatter plots of flow cytometric analyses for macrophages in the SVF of eWAT from mice given Veh or Clod for 2 (upper) and 6 (lower) weeks. (**c**) Percentage of living CD45^+^F4/80^+^ macrophages (ATMs) in the SVF of eWAT from mice given Veh or Clod for 2 (left) and 6 (right) weeks. (**d**–**g**) Relative expression levels of *Pdgfb* and *Vegfa* mRNA in eWAT from mice given Veh or Clod for 2 and 6 weeks. (**h**–**k**) Body and eWAT weights of mice after the 2- or 6-week administration protocol. Regarding the 2-week treatment, n = 7–9; for the 6-week treatment, n = 8–12. Data are shown as means ± S.E. *p < 0.05 and **p < 0.01, significantly different from Veh.

*Pdgfb* expression increases in CD45^+^F4/80^+^ATMs with obesity. Therefore, we investigated the impact of macrophage depletion on vascular remodeling in the adipose tissue of HFD-fed mice. We administered Clod to mice after 6 weeks of HFD feeding when *Pdgfb* gene expression markedly increases^11^. Mice were kept on HFD during 2 or 6 weeks of the Clod treatment (Fig. 1a). A flow cytometric analysis showed that Clod effectively depleted ATMs to approximately 10% in the living cells of SVF after the 2- and 6-week treatments (Fig. 1b,c). *Pdgfb* expression levels decreased in the eWAT of Clod-treated mice with both treatment periods (Fig. 1d,e). In contrast, *Vegfa* expression levels decreased in the eWAT of mice administered Clod for 6 weeks (Fig. 1f,g). Although neither body nor eWAT weights significantly changed in mice administered Clod for 2 weeks (Fig. 1h,i), they significantly decreased in those treated for 6 weeks (Fig. 1j,k) regardless of unchanged food consumption (data not shown).

**Figure 2:**
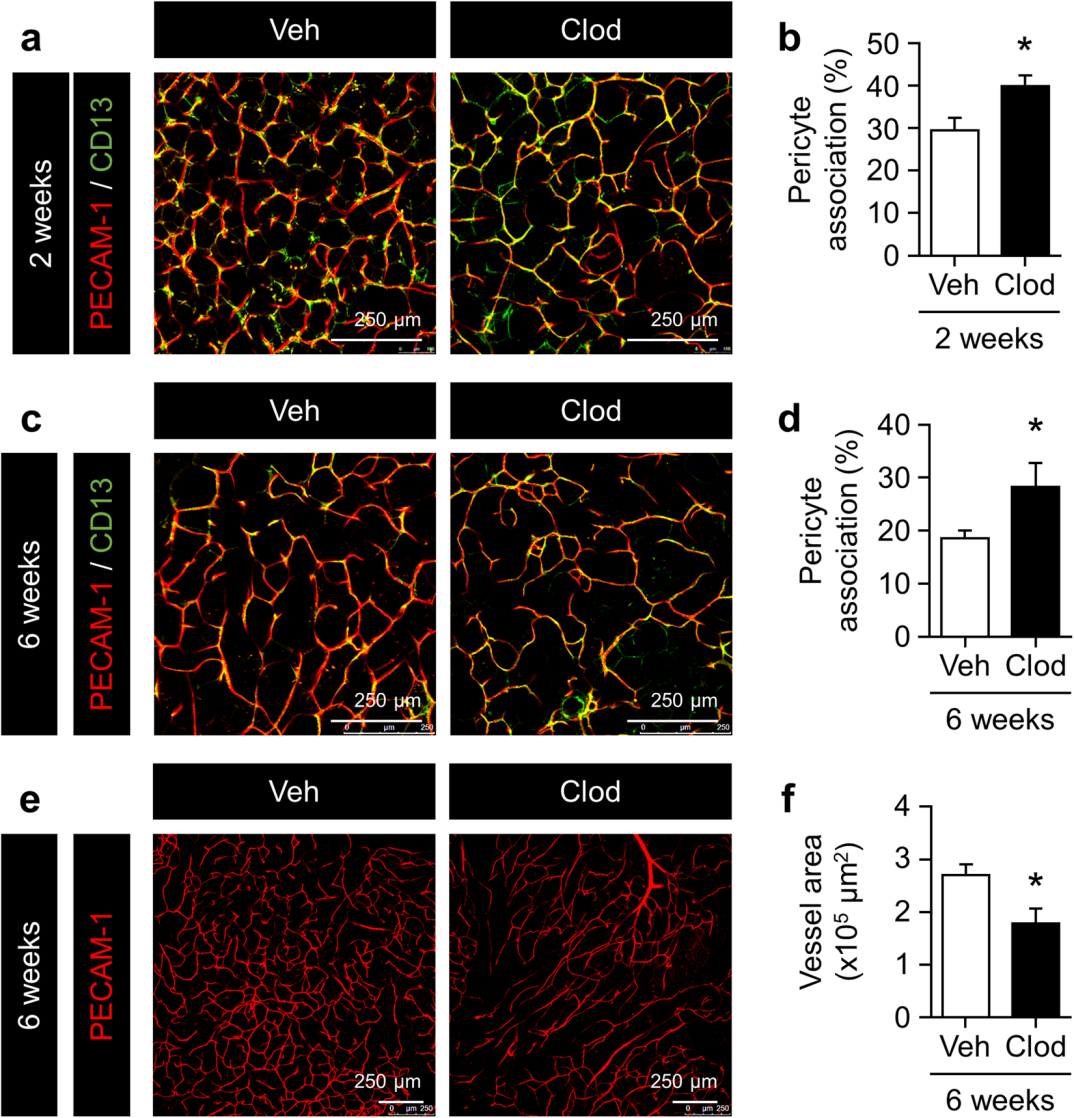
Impact of adipose macrophage deletion on pericyte coverage and vascular density in the eWAT of HFD-fed mice. (**a**,**c**) Representative images of pericytes and endothelial cells in eWAT from mice given Veh or Clod for 2 and 6 weeks. Pericytes and endothelial cells were stained with anti-CD13 (green) and anti-PECAM-1 (red) antibodies, respectively. (**b**,**d**) Percentage of the pericyte association with endothelial cells in eWAT from mice given Veh or Clod for 2 and 6 weeks. Percentages were quantified by the ratio of the associated area (yellow) to the endothelial area. (**e**,**f**) Representative images of endothelial cells stained with the anti-PECAM-1 antibody and quantified vessel area in the eWAT of mice given Veh or Clod for 6 weeks. Regarding the 2-week treatment, n = 7–9; for the 6-week treatment, n = 8–12. Data are shown as means ± S.E. *p < 0.05, significantly different from Veh.

Since few vessel-associated pericytes were observed in macrophage-accumulating areas in the eWAT of obese mice^11^, we performed whole-mount immunofluorescence on eWAT in these mice to investigate the impact of ATM deletion on pericyte associations in adipose vessels. CD13-positive pericytes rarely attached along vessels in the eWAT of control-treated mice in both treatment periods under HFD feeding (Fig. 2a,c). In contrast, pericytes tightly covered vessels in the eWAT of Clod-treated mice (Fig. 2a–d). Vessel areas significantly decreased in the eWAT of mice administered Clod for 6 weeks (Fig. 2e,f). These results suggest that ATMs contribute to adipose angiogenesis in obesity by promoting pericyte detachment from mature vessels.

### LPS and high-glucose condition augment *Pdgfb* expression in macrophages

**Figure 3:**
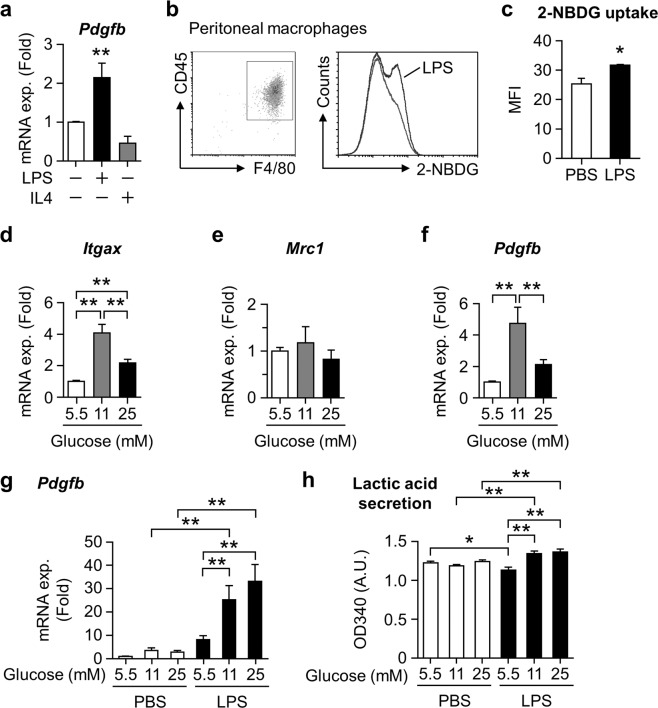
Impact of high glucose conditions on *Pdgfb* expression in macrophages. (**a**) *Pdgfb* mRNA expression levels in peritoneal macrophages treated with 100 ng/mL LPS or 10 ng/mL IL-4 under 11 mM glucose for 24 hours. n = 4 (**b**) Representative scatter plot of living CD45^+^F4/80^+^ peritoneal macrophages and a histogram of 2-NBDG in these cells by flow cytometry. (**c**) Relative changes in the mean fluorescent intensity (MFI) of 2-NBDG in living CD45^+^F4/80^+^ peritoneal macrophages treated with 100 ng/mL LPS for 24 hours. n = 3. (**d**–**f**) Relative expression levels of *Itgax*, *Mrc1*, and *Pdgfb* mRNA in RAW264.7 cells treated with several glucose concentrations for 24 hours. Mean of data among six independent experiments. n = 14–15. (**g**) Relative expression levels of *Pdgfb* mRNA in RAW264.7 cells treated with 100 ng/mL LPS for 3 hours under several glucose concentrations. Mean of data among three independent experiments. n = 6. (**h**) Relative lactic acid contents in the cultured medium of RAW264.7 cells treated with 100 ng/mL LPS for 3 hours under several glucose concentrations. Mean of data among three independent experiments. n = 4. Data are shown as means ± S.E. *p < 0.05 and **p < 0.01, among two groups, as indicated. A.U., arbitrary unit.

We investigated the mechanisms underlying PDGF-B production in the macrophages of obese adipose tissue. Since *Pdgfb* expression increases in ATMs with the progression of insulin resistance in obese mice^11^, the metabolic environment surrounding macrophages may have an impact on its expression. We examined the impact of the LPS or IL-4 stimulation, which directs macrophage polarity toward pro- or anti-inflammation, respectively, on *Pdgfb* expression in murine peritoneal macrophages (PMs). LPS, but not IL-4, augmented *Pdgfb* mRNA expression (Fig. 3a). Since activated macrophages require energy from the metabolites of glucose catabolism pathways to produce cytokines, we assessed glucose uptake using fluorochrome-conjugating glucose (2-NBDG) in CD45^+^F4/80^+^ PMs stimulated with LPS for 24 hours. The LPS treatment increased glucose uptake in macrophages (Fig. 3b,c), as previously reported^25^. We then treated RAW264.7 macrophages with higher glucose concentrations than hyperglycemia for 24 hours to investigate whether high glucose *per se* induces a pro-inflammatory profile. Macrophages exposed to high glucose selectively expressed high levels of *Itgax* mRNA encoding CD11c, expressed by pro-inflammatory macrophages, but not *Mrc1* encoding CD206, expressed by anti-inflammatory macrophages (Fig. 3d,e). Furthermore, *Pdgfb* mRNA expression levels were similarly increased by the high glucose treatment (Fig. 3f), suggesting that macrophages acquire the ability to induce *Pdgfb* expression by driving glucose catabolism. We investigated the glucose dependence of LPS-induced *Pdgfb* expression. High glucose exposure significantly augmented LPS-induced *Pdgfb* gene expression (Fig. 3g) in association with elevated lactic acid levels (Fig. 3h). Since the expression of toll-like receptor 4 (*Tlr4*) did not change with glucose concentrations (Fig. S1), glucose concentration dependent LPS-induced expression of *Pdgfb* is not regulated at the receptor expression level of TLR4. We also examined the impact of fatty acids as another source of metabolic stress on pro-inflammatory changes in macrophages with obesity^26^. However, the treatment with a fatty acid cocktail did not affect *Pdgfb* mRNA expression in Raw264.7 macrophages (data not shown). Therefore, increased glucose catabolism driven by pro-inflammatory activation is important for *Pdgfb* gene induction in macrophages.

### Glycolytic pathway is important for induction of inflammatory cytokines and *Pdgfb* in macrophages

**Figure 4:**
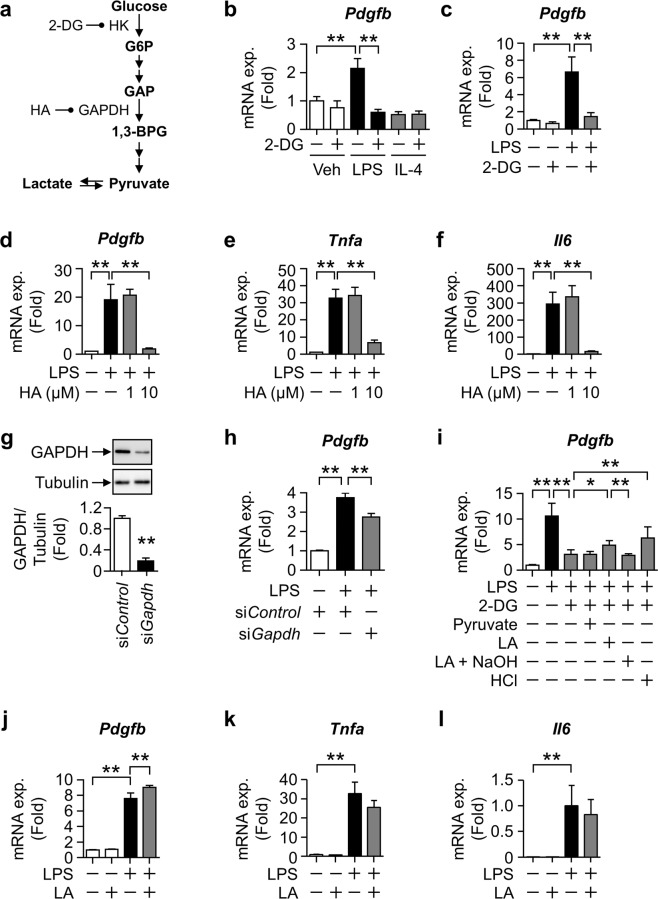
Importance of glycolysis for LPS-induced *Pdgfb* mRNA expression in macrophages. (**a**) Schematic pathway of glycolytic metabolism. The terminals of half lines represent the targets of inhibitors. (**b**) Relative expression levels of *Pdgfb* mRNA in peritoneal macrophages pretreated with 10 mM 2-DG for 0.5 hours and stimulated with 100 ng/mL LPS or 10 ng/mL IL-4 for 24 hours under 11 mM glucose. n = 4. (**c**–**f**) Relative expression levels of *Pdgfb*, *Tnfa*, and *Il6* mRNA in RAW264.7 cells pretreated with 2-DG or HA for 0.5 hours and stimulated with LPS for 3 hours under 11 mM glucose. Mean of data among three or four independent experiments. n = 6 or 5. (**g**) Representative images blotted with anti-GAPDH and anti-α-Tubulin antibodies, and relative signal density of GAPDH normalized with that of α-Tubulin in si*Gapdh*-transfected RAW264.7 cells. Mean of data among three independent experiments. n = 6. (**h**) Relative expression levels of *Pdgfb* mRNA in RAW264.7 cells stimulated with LPS for 3 hours under 11 mM glucose after si*Gapdh* transfection. Mean of data among three independent experiments. n = 6. (**i**) Relative expression levels of *Pdgfb* mRNA in RAW264.7 cells pretreated with 10 mM 2-DG for 0.5 hours, and stimulated with LPS for 3 hours under 11 mM glucose with the following co-treatment, as indicated: 20 mM pyruvate, 20 mM lactic acid (LA), 20 mM LA neutralized by 16.1 mM NaOH, or culture medium adjusted with 18.8 mM HCl. Mean of data among three independent experiments. n = 6. (**j**–**l**) Relative expression levels of *Pdgfb*, *Tnfa*, and *Il6* mRNA in RAW264.7 cells stimulated with LPS in the presence or absence of 20 mM LA for 3 hours under 11 mM glucose. Mean of data among five independent experiments. n = 5. Data are shown as means ± S.E. *p < 0.05 and **p < 0.01, among two groups, as indicated. HK, hexokinase; G6P, glucose-6-phosphate; GAP, glyceraldehyde 3-phosphate; GAPDH, glyceraldehyde phosphate dehydrogenase; 1,3-BPG, D-glycerate 1,3-bisphosphate; 2-DG, 2-deoxyglucose; HA, heptelidic acid.

We then investigated whether the glycolytic pathway contributes to *Pdgfb* induction in LPS-stimulated macrophages. Since glucose at 11 mM is sufficient to increase glycolytic activity in macrophages (Fig. 3g), we hereafter cultured cells in 11 mM glucose as the high glucose condition (HG). *Pdgfb* expression induced by HG plus the LPS stimulation was completely suppressed by 2-DG, an inhibitor of hexokinase (HK), which is an initial enzyme in glycolysis in PMs (Fig. 4a,b) and RAW264.7 cells (Fig. 4c) cultured under HG. Moreover, a treatment with heptelidic acid (HA), an inhibitor of GAPDH, abolished LPS-induced *Pdgfb* as well as *Tnfa* and *Il6* expression (Fig. 4d–f). Similar results were obtained in cells with siRNA-mediated GAPDH knockdown (Fig. 4g,h). The down-regulated expression of *Pdgfb* by a pre-treatment with 2-DG was recovered by lactic acid and extracellular acidification adjusted with hydrochloride (HCl), but not by supplementation with pyruvate. Furthermore, the effects of lactic acid were canceled by neutralization using sodium hydrate (NaOH) (Fig. 4i). Lactic acid also augmented the LPS-induced expression of *Pdgfb* without any changes in that of inflammatory cytokines (Fig. 4j–l). These results indicate that glycolysis is essential for the induction of *Pdgfb* expression as well as proinflammatory cytokines in macrophages. Nevertheless, the impact of lactic acid from glycolysis on the induction of *Pdgfb* was distinct from that of inflammatory cytokines in macrophages.

### Divergent signaling pathways for gene induction from TLR4 signaling-coupled glycolytic metabolism in macrophages

**Figure 5:**
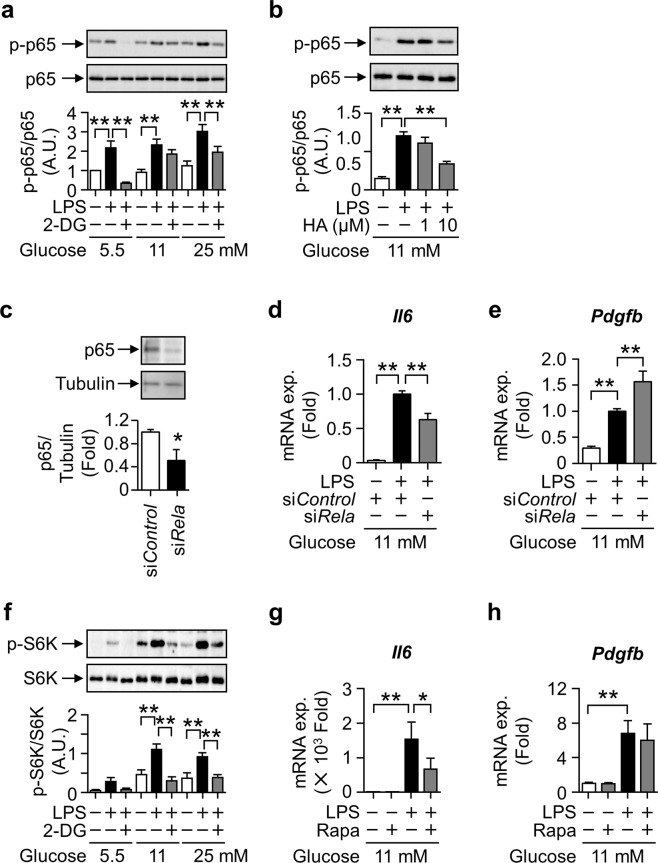
Relevance of p65 and mTORC1 signaling pathways to LPS-induced *Pdgfb* mRNA expression coupled with glycolysis in macrophages. (**a**) Representative images blotted with anti-phospho-p65 and anti-p65 antibodies, and relative signal density of phospho-p65 normalized with that of p65 in RAW264.7 cells pretreated with 10 mM 2-DG for 0.5 hour and stimulated with 100 ng/mL LPS for 0.5 hours. Mean of data among four independent experiments. n = 4. Statistical analyses were performed under the same glucose concentration. (**b**) Representative images blotted with anti-phospho-p65 and anti-p65 antibodies, and relative signal density of phospho-p65 normalized with that of p65 in RAW264.7 cells pretreated with HA for 0.5 hours and stimulated with 100 ng/mL LPS for 3 hours. Mean of data among three independent experiments. n = 4. (**c**) Representative images blotted with anti-p65 and anti-α-Tubulin antibodies, and relative signal density of p65 normalized with that of α-Tubulin in si*Rela*-transfected RAW264.7 cells. Mean of data among three independent experiments. n = 6. (**d**,**e**) Relative expression levels of *Il6* and *Pdgfb* mRNA in RAW264.7 cells stimulated with 100 ng/mL LPS for 3 hours after si*Rela* transfection. Mean of data among three independent experiments. n = 6. (**f**) Representative images blotted with anti-phospho-S6K and anti-S6K antibodies, and relative signal density of phospho-S6K normalized with that of S6K in RAW264.7 cells pretreated with 10 mM 2-DG for 0.5 hours and stimulated with 100 ng/mL LPS for 3 hours. Mean of data among four independent experiments. n = 4. Statistical analyses were performed under the same glucose concentration. (**g**,**h**) Relative expression levels of *Il6* and *Pdgfb* mRNA in RAW264.7 cells pretreated with 10 nM rapamycin (Rapa) for 1 hour and stimulated with 100 ng/mL LPS for 3 hours. Mean of data among four independent experiments. n = 8. Data are shown as means ± S.E. *p < 0.05 and **p < 0.01, among two groups, as indicated. A.U., arbitrary unit.

The expression of *Pdgfb* and inflammatory cytokines was suppressed by the inhibition of GAPDH, implying the existence of a common transcriptional pathway promoting their expression. Therefore, we investigated the transcriptional machinery of *Pdgfb* by glycolysis in LPS-treated macrophages. Since *Pdgfb* promoter regions have a κB site^27^, we assessed the involvement of NFκB. The LPS-induced phosphorylation of the NFκB p65 subunit was significantly attenuated by the 2DG and HA pretreatments (Fig. 5a,b). The LPS-induced expression of *Il6* was attenuated by the knockdown of p65 (Fig. 5c,d), as previously reported^28^. However, the expression of *Pdgfb* was not suppressed in p65-knockdown macrophages (Fig. 5e).

Previous studies demonstrated that the activation of mTORC1 regulates macrophage production of proinflammatory cytokines^19,29^. To clarify the signal transduction pathways mediating glycolysis to induce *Pdgfb* expression in LPS-stimulated macrophages, we evaluated the phosphorylation of S6 Kinase (S6K), a substrate of mTORC1. The phosphorylation of S6K was increased by the LPS stimulation in RAW264.7 cells under 11 and 25 mM glucose conditions, and was suppressed by 2-DG (Fig. 5f). A treatment with rapamycin, an inhibitor of mTORC1, suppressed *Il6* expression in macrophages stimulated with LPS under HG (Fig. 5g). However, the rapamycin treatment did not affect *Pdgfb* expression (Fig. 5h), indicating that the systems for *Pdgfb* mRNA induction differ from the mTORC1 pathway.

**Figure 6:**
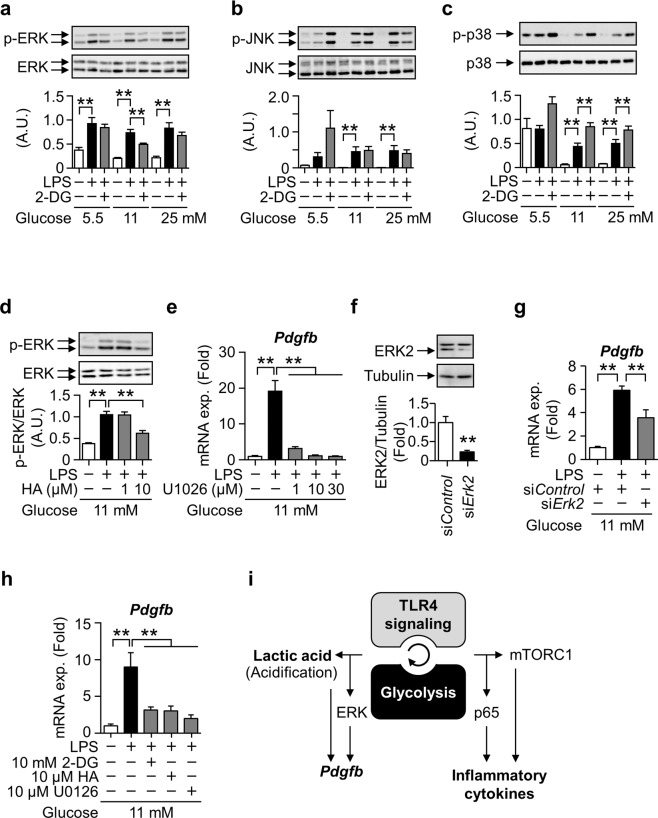
Relevance of MAPK signaling pathways to LPS-induced *Pdgfb* mRNA expression coupled with glycolysis in macrophages. (**a**-**c**) Representative images blotted with anti-phospho-ERK1/2, anti-ERK, anti-phospho-JNK, anti-JNK, anti-phospho-p38, and anti-p38 antibodies, and relative signal density of phosphorylated protein normalized with that of each total protein in RAW264.7 cells pretreated with 10 mM 2-DG for 0.5 hours and stimulated with 100 ng/mL LPS for 3 hours. Mean of data among four independent experiments. n = 4. Statistical analyses were performed among the same glucose concentration. (**d**) Representative images blotted with anti-phospho-ERK1/2 and anti-ERK1/2 antibodies and the relative signal density of phospho-ERK2 normalized with that of ERK2 in RAW264.7 cells pretreated with HA for 0.5 hours and stimulated with 100 ng/mL LPS for 3 hours. Mean of data among three independent experiments. n = 4. (**e**) Relative expression levels of *Pdgfb* mRNA in RAW264.7 cells pretreated with U0126 for 2 hours and stimulated with 100 ng/mL LPS for 3 hours. Mean of data among three independent experiments. n = 6. (**f**) Representative images blotted with anti-ERK1/2 and anti-α-Tubulin antibodies, and the relative signal density of ERK2 normalized with that of α-Tubulin in si*Erk2-*transfected RAW264.7 cells. Mean of data among three independent experiments. n = 3. (**g**) Relative expression levels of *Pdgfb* mRNA in RAW264.7 cells stimulated with 100 ng/mL LPS for 3 hours after the si*Erk2* transfection. Mean of data among six independent experiments. n = 6. (**h**) Relative expression levels of *Pdgfb* mRNA in BMDMs pretreated with 2-DG or HA for 0.5 hours, or U0126 for 2 hours and stimulated with 100 ng/mL LPS for 3 hours. n = 4. Data are shown as means ± S.E. *p < 0.05 and **p < 0.01, among two groups, as indicated. (**i**) A schematic illustration of divergent signaling pathways for gene induction from TLR4 signaling-coupled glycolytic metabolism in macrophages. Synergy promotes the induction of *Pdgfb* and inflammatory cytokine genes. *Pdgfb* mRNA expression is induced through the ERK signaling pathway and extracellular acidosis caused by lactic acid production, whereas that of inflammatory cytokine genes is induced through p65 NFκB and mTORC1. A.U., arbitrary unit; TLR4, toll-like receptor 4; mTORC1, mechanistic target of rapamycin complex 1.

The mitogen-activated protein kinase (MAPK) family plays a role in the regulation of pro-inflammatory macrophages^30^. Therefore, we examined the significance of these kinases in the glycolysis-dependent induction of *Pdgfb* expression in LPS-stimulated macrophages. The LPS treatment stimulated the phosphorylation of ERK, JNK, and p38 independently of glucose concentrations. The 2-DG treatment suppressed the LPS-stimulated phosphorylation of ERK among MAPKs under HG (Fig. 6a–c). The HA treatment also suppressed the LPS-stimulated phosphorylation of ERK (Fig. 6d). The treatment with U0126, an inhibitor of ERK, as well as ERK2 knockdown significantly suppressed LPS-induced *Pdgfb* expression (Fig. 6e–g). These results indicate that the glycolysis-coupled ERK pathway mediates the induction of *Pdgfb* in macrophages. Since the ERK inhibitor did not significantly affect the phosphorylation of p65 (Fig. S2a,b), the activation of p65 NFκB was independent of ERK activation under our experimental conditions. LPS-induced *Pdgfb* expression was also observed in non-polarized bone marrow-derived macrophages (BMDMs), which was again inhibited by pretreatments of 2DG, HA, and U1026 (Fig. 6h). Therefore, the induction mechanism through TLR4 signaling coupled with glycolysis appears to be common in both unstimulated and inflammatory macrophages.

## Discussion

WAT expands with remodeling of the existent vascular network. In the present study, we demonstrated that pericyte detachment from matured blood vessels, an initial step in remodeling, was regulated by PDGF-B derived from infiltrated pro-inflammatory macrophages in obesity. The metabolic stresses of inflammation and high-glucose stimuli promote glycolysis in macrophages, and increase PDGF-B expression through the glycolysis-coupled activation of the ERK pathway. Furthermore, stromal acidosis resulting from lactic acid within hypoxic obese adipose tissue appears to be involved in *Pdgfb* mRNA expression in macrophages.

Obesity-related insulin resistance is exacerbated by adipose tissue hypertrophy with angiogenesis. New branches sprouting from pre-existing blood vessels require the dissociation of pericytes that cover and inhibit of endothelial cell proliferation^7–10^. Serial angiogenic processes during tissue remodeling are regulated by macrophages^5,31^. Diet-induced body weight gain positively correlates with CD11c expression in eWAT, which has a higher angiogenic capacity than inguinal WAT^32^. However, whether ATMs contribute to adipose angiogenesis during obesity remains unclear. We previously demonstrated that excessive PDGF-B detaches pericytes from vessels through PDGFRβ, resulting in angiogenesis in WAT during obesity^11^. Moreover, PDGF-B is strongly expressed in pro-inflammatory CD11c^+^ATMs^11^. In the present study, we demonstrated that PDGF-B expression and pericyte detachment were diminished by the depletion of ATMs in the eWAT of diet-induced obese mice. Therefore, we concluded that infiltrated pro-inflammatory ATMs produce excessive PDGF-B, which stimulates pericyte dissociation from vessels, resulting in the promotion of neoangiogenesis during diet-induced obesity.

Vascular rarefaction and increased oxygen consumption in adipocytes cause hypoxia in interstitium of obese WAT^33,34^. Pro-inflammatory ATMs preferably accumulate in these hypoxic interstitium^13^, induce chronic inflammation in a hypoxia-inducible factor (HIF) 1α-dependent manner, resulting in impaired blood flow during obesity^35^, and it forms a vicious circle. Consistent with a previous report showing that loss of pericyte coverage causes hypoperfusion^36^, current study demonstrated that excessive PDGF-B secreted from infiltrating ATMs dissociates pericytes from vessels. Taken together, the hypoperfusion with PDGF-B induced pericyte detachment and HIF1α activation could explain the mechanism of unhealthy adipose tissue expansion^11^.

The expression of *Vegfa* did not decrease in eWAT depleted ATMs 2 weeks after Clod injection, whereas *Vegfa* expression was significantly low in accordance with less vascularity and lower eWAT weight in the eWAT of mice administered Clod for 6 weeks (Figs. 1 and 2). Since pro-inflammatory activated macrophages promote *Vegfa* expression in adipocytes^37^, low *Vegfa* expression was attributed to the remission of inflammation by ATM depletion. These findings suggest a mechanism that PDGF-B secreted from ATMs detaches pericytes and VEGF-A secreted from adipocytes elongates the vasculature in a serial angiogenic process in eWAT during obesity. In contrast, adipocyte-derived VEGF-A plays an important role in healthy expansion of adipose tissue by neovascularization^1,2,38^. In this regard, HIF1α-positive inflammatory ATMs suppress expression of angiogenic factors including VEGF-A in preadipocytes and endothelial cells, leading to vascular rarefaction in unhealthy expansion of adipose tissue^35^. In any case, further analyses focusing on the adipose angiogenesis after pericyte detachment are necessary for further understanding of angiogenic mechanism in obesity that determines the fate with healthy or unhealthy expansion.

Activated macrophages reprogram their metabolic properties in response to various stimuli^17^. The pro-inflammatory changes induced by the activation of TLR4 signaling drives glycolysis, and vice versa^17^. However, the interplay between intracellular metabolism and signal transduction pathways relevant to their functions in macrophages remains unclear. We revealed that the system of *Pdgfb* induction is distinct from that of inflammatory cytokines depending on glycolytic activity in LPS-activated macrophages (Fig. 6i). TLR4 signaling-coupled glycolysis activates the ERK, NFκB, and mTORC1 pathways, with only the ERK pathway being involved in the induction of PDGF-B. An acidic environment due to lactic acid appears to up-regulate PDGF-B expression. In contrast, the glycolysis-stimulated NFκB and mTORC1 pathways did not affect *Pdgfb* expression, but played significant roles in the inflammatory responses of macrophages, as reported previously^29^.

Hypoxia increases lactic acid production by anaerobic metabolism^39^. Lactic acid levels in WAT were shown to be higher in obese mice than in lean mice^39,40^. The present results demonstrated that LPS-activated glycolysis stimulated lactic acid production, which was more evident under HG (Fig. 3h). Furthermore, *Pdgfb* expression was augmented by the stimulation with lactic acid and acidic conditions *in vitro* (Fig. 4i,j). Since lactic acid promotes local tissue acidification in the interstitium of obese WAT, an acidic environment may potentiate *Pdgfb* induction in macrophages. Elevations in lactic acid levels were observed in the WAT, but not skeletal muscle, of obese mice^39^. This may partly explain previous findings showing that *Pdgfb* expression only increased in WAT among the insulin-target tissues of obese mice^11^, even though pro-inflammatory macrophages accumulate in several peripheral tissues^41^.

In conclusion, the present study provides an insight into the novel role of macrophages in vascular remodeling during obesity. Obesity-related triple distress, namely, adipose hypertrophy, hyperglycemia, and chronic inflammation, exacerbates PDGF-B production in pro-inflammatory macrophages, resulting in pericyte detachment towards angiogenesis within WAT during obesity. These results indicate the significance of macrophage-derived PDGF-B as a potential therapeutic target of obesity-related insulin resistance and metabolic disorders.

## Materials and Methods

### Reagents

The reagents used in the present study were listed in Supplemental Table 1.

### Preparation of liposome-encapsulated clodronate

Three-sn-phosphatidylcholine (from egg yolk) 172 mg, cholesterol 16 mg, and α-tocopherol 0.5 mg were dissolved in 2 mL of methanol-chloroform solution (methanol: chloroform = 1: 2) in a round-bottomed flask. After evaporating organic solvents, the lipid film was hydrated with 1 mL of 200 mg/mL disodium clodronate tetrahydrate (Tokyo Chemical Industry, Tokyo, Japan) or 9.57 mM PBS for the vehicle control. Suspensions were freeze-thawed 5 times by transferring between liquid nitrogen and a water bath at 40 °C to increase the inclusion rate in liposomes^42^. Liposomes were extruded 21 times through a 0.4-μm pore polycarbonate filter using a LiposoFast extruder (Avestin, Mannheim, Germany). Liposome sizes obtained by a FPAR-1000 particle analyzer (Otsuka Electronics, Osaka, Japan) were confirmed to be 293.4 ± 19.6 nm (clodronate) or 555.9 ± 57.2 nm (PBS). The encapsulating rate of clodronate in liposomes was estimated to be 70%, as previously described^42^. Suspensions were stored at 4 °C and used within 2 weeks.

### Animals

All animal experiments followed institutional guidelines for the use and care of laboratory animals and were approved by the University of Toyama Ethics Committee. Mice were maintained at 20–26 °C with normal chow diet (PicoLab Rodent Diet, LabDiet, St. Louis, MO, U.S.A) and water available *ad libitum* in a 12-hour light/dark cycle-controlled room with a specific pathogen-free environment. Male C57BL/6 J mice were fed 60 kcal% HFD (D12492; Research Diet, New Jersey, U.S.A) from 8–11 weeks old for 8 or 12 weeks. Regarding the administration of liposome-encapsulated clodronate (Clod) or PBS (Veh), stocked liposome suspensions were centrifuged at 21,130 × *g* and 4 °C for 20 min and saved pellets were resuspended in PBS at 10%. Mice were intraperitoneally administered the liposome suspension (0.1 mL/10 g body weight, an estimated 140 mg/kg clodronate) twice a week for 2 or 6 weeks after 6 weeks of HFD.

### Flow cytometric analysis

To separate the stromal vascular fraction (SVF) from the epididymal WAT (eWAT) of mice, minced eWAT was digested in KRHAG buffer (2% bovine serum albumin (BSA), 4.6 mM KCl, 2.3 mM CaCl_2_, 1.14 mM KH_2_PO_4_, 1.14 mM MgSO_4_, 32 mM HEPES, and 2 mM glucose in saline) containing 2 mg/mL collagenase and 50 μg/mL DNase I at 37 °C for 45 min. After centrifugation at 1,500 rpm and 4 °C for 10 min, cell pellets as SVF were washed twice with KRHAG buffer. SVF was hemolyzed with lysing buffer at 4 °C for 15 min. SVF was washed twice with FACS buffer consisting of 1% BSA in FACS Sheath Flow followed by filtration through a 190-μm nylon mesh. SVF was stained with fluorochrome-conjugated antigen-specific antibodies or their isotype control antibodies on ice for 30 min after blocking with the anti-CD16/CD32 antibody on ice for 10 min. After washing once with FACS buffer, cell suspensions were stained with 7AAD just before sorting. The total amount of SVF from samples was sorted by FACS Canto II (BD Biosciences, New Jersey, U.S.A). The percentage of macrophages in living cells, excluding cell doublets, was analyzed by FCS Express4.0 (De Novo Software, California, U.S.A.).

### Real-time PCR

Total RNA was extracted from the dissected eWAT of mice and cultured cells with TRIsure using the following procedure. Equal amounts of total RNA were subjected to RT-PCR using the PrimeScript™ RT reagent Kit. Gene expression was analyzed by real-time PCR with 0.2 μM of primers listed in Supplemental Table 2 and TB Green™ Premix Ex Taq™ II using Mx3000/3005 P (Agilent, California, U.S.A). The expression level of the target gene was normalized to that of the *Rn18s* gene.

### Whole-mount immunofluorescence

Whole-mount immunofluorescence was performed as described previously^11^. Dissected eWAT was fixed with 1% paraformaldehyde at 4 °C overnight and then washed with 9.57 mM PBS three times for 15 min each. Trimmed tissue samples (height 5 mm × width 5 mm) were permeabilized with 20 μg/mL proteinase K in 10 mM Tris-HCl buffer at room temperature for 5 min. Samples were washed with PBS containing 0.03% tween 20 (PBS-T) three times at room temperature for 20 min each, following blocking with a protein block at 4 °C overnight. After washing six times with PBS-T, samples were incubated with a primary antibody-containing Antibody Diluent at 4 °C overnight. After washing six times with PBS-T, samples were blocked with the protein block at room temperature for 2 hours. Samples were incubated in secondary antibody-containing Antibody Diluent at 4 °C overnight, followed by washing six times with PBS-T. Samples were incubated in clearing reagents to reduce scattering light, as described previously^43^. The fluorescent signals of samples on a glass-bottomed dish were randomly observed by confocal microscopy (Leica TCS SP5, Wetzlar, Germany). To evaluate vessel areas, the fluorescent intensity of CD31 was measured by ImageJ. Regarding the pericyte association along vessels, merged areas with CD13 and CD31 were measured by the plugin RG2B Colocalization in ImageJ and divided by vessel areas. Signal intensities were equally processed among samples by Paint.NET (V3.10).

### Cell culture

Peritoneal macrophages (PMs) were collected from the abdominal cavities of 8-week-old male C57BL/6 J mice 3 days after an intraperitoneal injection of 4% thioglycollate. Adherent cells were used as PMs in the first 2 hours after seeding on plates. PMs seeded at 1 × 10^6^ cells/well on 24-well plates and cultured in RPMI containing 10% fetal bovine serum (FBS) for 18–22 hours were used in analyses of gene expression, while those seeded at 0.5 or 1.0 × 10^6^ cells/well on 6-well plates and cultured in medium for 34–39 hours were used in analyses of glucose uptake. PMs were starved in serum-free DMEM containing 5.56 mM glucose (LG-DMEM) for 12–13 hours. PMs were treated with or without 10 mM 2-deoxy D-glucose (2-DG) for 0.5 hours before 100 ng/mL LPS, 10 ng/mL IL-4, or the vehicle treatment on 11 mM glucose for 24 hours.

RAW264.7 cells were maintained in DMEM containing 25 mM glucose (HG-DMEM) and 10% FBS on 100-mm dishes and passaged every 2–3 days. RAW264.7 cells were seeded at 2.63 × 10^5^ cells/cm^2^ on 12- and 24-well plates in HG-DMEM containing 10% FBS for one day, followed by starvation in serum-free LG-DMEM for 12–16 hours. RAW264.7 cells were treated with various inhibitors for 0.5 hours, rapamycin (Rapa) for 1 hour, or U0126 for 2 hours, and stimulated with LPS (100 ng/mL) for the indicated times. Glucose concentrations in media were changed to between 5.5 and 25 mM, and mannitol was added to media to balance osmolality among different glucose concentrations.

BMDMs were differentiated from bone marrow cells isolated from the femur and tibia of male C57BL/6 J mice, by using RPMI supplemented with 10% FBS and 20% L929 condition medium, as described previously^44^. PMs and RAW264.7 cells were cultured in a humidified chamber at 37 °C under 5% CO_2_.

### siRNA transfection

RAW264.7 cells seeded at 2 × 10^4^ cells/well were cultured in HG-DMEM containing 10% FBS in 24-well plates overnight. Lipid complexes with 0.375% Lipofectamine^®^ RNAiMAX and the siRNA of the negative control, *Gapdh* (50 nM si*Gapdh*), *Erk2* (50 nM si*Erk2*), or *Rela* (100 nM si*Rela*) in Opti-MEM^®^ were added to HG-DMEM with 0.1% FBS and antibiotics in plate wells. Media were replaced with HG-DMEM containing 0.1% FBS and antibiotics after 6 (si*Erk2*) or 24–27 hours (si*Gapdh* and si*Rela*). si*Gapdh*-transfected cells were supplemented with 10 mM sodium pyruvate. After culturing for a further 14 (si*Erk2*) or 32 hours (si*Gapdh* and si*Rela*), cells were serum starved for 13 hours in LG-DMEM and then stimulated with 100 ng/mL LPS under 11 mM glucose for 3 hours.

### Glucose uptake assay and measurement of lactic acid contents

PMs were treated with 100 ng/ml LPS for 24 hours. Fluorochrome-conjugated glucose 2-NBDG (30 μM) was added 23 hours after LPS stimulation. PMs were stained with PE/Cy7 anti-CD45 and APC/Cy7 anti-F4/80 antibodies for 30 min after blocking with the anti-CD16/CD32 antibody on ice for 10 min. Cell suspensions were washed twice and then stained with 7AAD 10 min before sorting to FACS Canto II. Lactic acid contents in cultured media were assessed as described previously^45^.

### SDS-PAGE and western blotting

In the preparation of protein lysates, cells were lysed with lysing buffer (4.2 g/L NaF, 50 mM Tris, 5 mM EDTA, 10 mM Na_4_P_2_O_7_, 0.58% aprotinin, 10 μg/mL leupeptin, 2 mM Na_3_VO_4_, 1% Triton X-100, 150 mM NaCl, 0.5% sodium deoxycholate, and 0.1% sodium dodecyl sulfate). Protein concentrations in lysates were measured using the Bradford assay. Equal concentrations of protein-containing lysates were mixed with a half volume of Laemmli sample buffer (0.01% bromophenol blue, 50 mM sodium phosphate, 50% glycerol, and 10% sodium dodecyl sulfate), followed by boiling for 5 min.

Western blotting was performed as described previously^46^. Chemiluminescence was observed on membranes using ImageQuant LAS4000mini (GE Healthcare Japan, Tokyo, Japan), following an incubation in chemiluminescent reagent Chemi Lumi One L. Specific signal densities were measured by ImageJ 1.50i (National Institutes of Health, USA).

### Statistical analyses

Data show means ± S.E. Statistical analyses were performed with the Student’s *t*-test between two groups or a one-way ANOVA followed by Bonferroni’s test for multiple comparisons using the software ystat2004. P < 0.05 was considered significant.

## Supplementary information

Supplementary information.
