# Supplementary material for: Pro-inflammatory macrophages coupled with glycolysis remodel adipose vasculature by producing platelet-derived growth factor-B in obesity
Source: Sci Rep. 2020 Jan 20;10:670. doi: 10.1038/s41598-019-57368-w (PMC6970998; doi:10.1038/s41598-019-57368-w)
Supplement: Supplementary file 1 — Supplementary information. [file 41598_2019_57368_MOESM1_ESM.pdf]

Supplementary Information

Pro-inflammatory macrophages coupled with glycolysis remodel adipose vasculature by producing platelet-derived growth factor-B in obesity

Yasuhiro Onogi[1], Tsutomu Wada[1*], Akira Okekawa[1], Takatoshi Matsuzawa[1], Eri Watanabe[1], Keisuke Ikeda[2], Minoru Nakano[2], Munehiro Kitada[3], Daisuke Koya[3], Hiroshi Tsuneki[1], Toshiyasu Sasaoka[1*].

[1]Department of Clinical Pharmacology, [2]Department of Biointerface Chemistry, University of Toyama, 2630 Sugitani, Toyama, Japan
[3]Department of Internal Medicine, Kanazawa Medical University, 1-1 Daigaku, Uchinada, Ishikawa 920-0293, Japan.

[*]Corresponding authors:
Tsutomu Wada and Toshiyasu Sasaoka
Department of Clinical Pharmacology, University of Toyama
2630 Sugitani, Toyama, 930-0194, Japan
Tel.: +81-76-434-7514, Fax: +81-76-434-5067
E-mail: twada@pha.u-toyama.ac.jp (TW),
tsasaoka@pha.u-toyama.ac.jp (TS)

Supplementary Table 1.
List of reagents used in the present study
Supplementary Table 2.
Primer List

Supplementary Figure 1.
Tlr4 mRNA expression in RAW264.7 cells.
Supplementary Figure 2.
The relevance of MAPK signaling pathways to the phosphorylation of p65 NFκB in LPS-stimulated macrophages.
Supplementary Figure 3.
Original full length western blot of Figure 4g.
Supplementary Figure 4.
Original full length western blot of Figure 5.
Supplementary Figure 5.
Original full length western blot of Figure 6.

Supplementary Table 1

Reagent	Source	Identifier
**Preparation for liposome-encapsulated clodronate**		
3-sn-phosphatidylcholine	FUJIFILM Wako Pure Chemical Corporation	169-21183
Cholesterol	FUJIFILM Wako Pure Chemical Corporation	034-03002
Disodium clodronate tetrahydrate	Tokyo Chemical Industry	D4160
α-tocopherol	FUJIFILM Wako Pure Chemical Corporation	207-01792
**Flow cytometric analysis**		
2-NBDG	Peptide Institute	23002-v
7AAD	Thermo Fisher Scientific	00-6993-50
anti-CD16/CD32 antibody	Thermo Fisher Scientific	14-0161-85
APC/Cy7 anti-F4/80 antibody	BioLegend	123118
APC/Cy7 isotype control	BioLegend	400524
Collagenase	FUJIFILM Wako Pure Chemical Corporation	032-22364
DNase I	Sigma	DN25
PE/Cy7 anti-CD45 antibody	Thermo Fisher Scientific	25-0451-82
**Real-time PCR**		
RrimeScript™ RT reagent Kit	Takara	RR037A
TB Green™ Premix Ex Taq™ II	Takara	RR820L
TRIsure	Bioline	BIO-38032
**Whole-mount immunofluorescent staining**		
Anti-Armenian Hamster IgG, Cy3 counjugate	Jackson ImmunoResearch	127-165-160
Antibody Diluent	Agilent	S2022
Anti-CD13 antibody	BIO-RAD	MCA2395
Anti-PECAM1 antibody	Merck Millipore	MAB1398Z
Anti-rat IgG, Alexa488 conjugate	Thermo Fisher Scientific	A-11006
Protein block, Serum-Free, Liquid form	Agilent	X0909
**Cell culture**		
DMEM, High Glucose	Thermo Fisher Scientific	12100046
DMEM, Low Glucose, Pyruvate	Thermo Fisher Scientific	31600034
Fetal bovine serum	Sigma	172012-500ML
Lipofectamine® RNAiMAX Transfection Reagent	Thermo Fisher Scientific	13778030
Opti-MEM® I Reduced Serum Medium	Thermo Fisher Scientific	31985062
RPMI 1640 Medium	Thermo Fisher Scientific	31800022
Thioglycollate	BD	211716
**Stimulant/Inhibitors**		
2-deoxy D-glucose	Tokyo Chemical Industry	D0051
Heptelidic acid	Cayman Chemical	14079
IL-4, Murine, Recombinant	Pepro Tech Inc.	214-14
Lactic acid	nacalai tesque	598-82-3
Lipopolysaccharides from *E.Coli*	Sigma	L6529
Rapamycin	Cell Signaling Technology	#9904S
Sodium Pyruvate	FUJIFILM Wako Pure Chemical Corporation	199-03062
U0126	abcam	ab120241
**Lactic acid content**		
Lactic dehydrogenase	FUJIFILM Wako Pure Chemical Corporation	300-52721
NAD^+	FUJIFILM Wako Pure Chemical Corporation	049-16461
**Western blotting**		
Anti-ERK1/2 antibody	Cell Signaling Technology	#9102
Anti-GAPDH antibody	Merck Millipore	MAB374
Anti-Goat IgG, HRP conjugate	Santa Cruz Biotechnology	sc-2020
Anti-JNK antibody	Santa Cruz Biotechnology	sc-571
Anti-Mouse IgG, HRP conjugate	GE Healthcare	NA931
Anti-NFκB p65 antibody	Santa Cruz Biotechnology	sc-372-G
Anti-p38α antibody	Santa Cruz Biotechnology	sc-535
Anti-p70 S6 Kinase antibody	Cell Signaling Technology	#9202
Anti-phospho-ERK1/2 (Thr202/Tyr204) antibody	Cell Signaling Technology	#9101
Anti-phospho-NFκB p65 (Ser536) antibody	Cell Signaling Technology	#3033
Anti-phospho-p38 MAPK (Thr180/Tyr182) antibody	Cell Signaling Technology	#9211
Anti-phospho-p70 S6 Kinase (Thr389) antibody	Cell Signaling Technology	#9205
Anti-phospho-SAPK/JNK (Thr183/Tyr185) antibody	Cell Signaling Technology	#4668
Anti-Rabbit IgG, HRP conjugate	GE Healthcare	NA934
Anti-α-Tubulin antibody	Cell Signaling Technology	#2125
Can Get Signal Solusion 1 & 2	TOYOBO	NKB-101
Chemi Lumi One L	nacalai tesque	07880-70
Prestained SDS-PAGE Standards, broad range	BIO-RAD	#1610318
WIDE-VIEW™ Prestained Protein Size Marker III	FUJIFILM Wako Pure Chemical Corporation	230-02461

Supplementary Table 2

Primer List		
Genes	Forward primer	Reverse primer
Il6	ATGGATGCTACCAAACTGGAT	TGAAGGACTCTGGCTTTGTCT
Itgax	ATGTTGGAGGAAGCAAATGG	CCTGGGAATCCTATTGCAGA
Mrc1	TGTGGTGAGCTGAAAGGTGA	CAGGTGTGGGCTCAGGTAGT
Pdgfb	CCCACAGTGGCTTTTCATTT	GTGAACGTAGGGGAAGTGGA
Rn18s	GTAACCCGTTGAACCCCATT	CCATCCAATCGGTAGTAGCG
Tlr4	CCTGGCTGGTTTACACGTC	GACATTGCAGAAACATTCGC
Tnfa	AAGCCTGTAGCCCACGTCGTA	GGCACCACTAGTTGGTTGTCTTTG
Vegfa	GAAGGAGAGCAGAAGTCCCA	ACACAGGACGGCTTGAAGAT
siRNA	Sense Strand (5'→3')	Antisense Strand (5'→3')
si*Control*	GUACCGCACGUCAUUCGUAUC	UACGAAUGACGUGCGGUACGU
si*Erk2*	GCUCUUGAAGACACAGCACUU	GUGCUGUGUCUUCAAGAGCUU
si*Gapdh*	CGGGAAGCUCACUGGCAUGUU	CAUGCCAGUGAGCUUCCCGUU
si*Rela*	GAUCAAUGGCUACACAGGAUU	UCCUGUGUAGCCAUUGAUCUU

Supplementary Figure 1

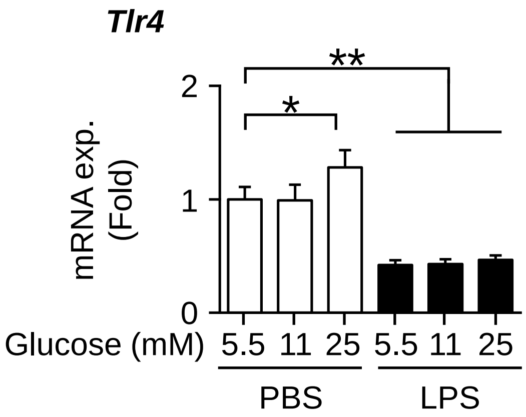

Supplementary Figure 1.

***Tlr4* mRNA expression in RAW264.7 cells.** Relative expression levels of *Tlr4* mRNA in RAW264.7 cells treated with 100 ng/mL LPS for 3 hours under several glucose concentrations. Mean of data among three independent experiments. n = 6. Data are shown as means ± S.E. *p<0.05 and **p<0.01, among two groups, as indicated.

Supplementary Figure 2

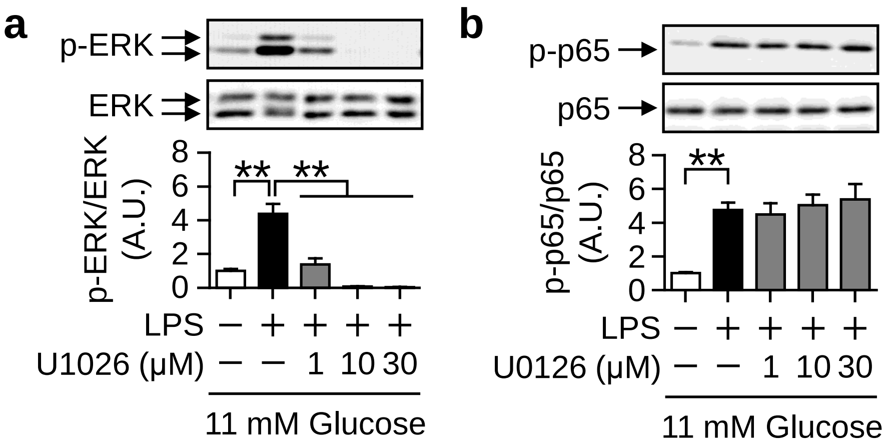

Supplementary Figure 2.

The relevance of MAPK signaling pathways to the phosphorylation of p65 NFκB in LPS-stimulated macrophages. (a and b) Representative images blotted with anti-phospho-ERK1/2, anti-ERK1/2, anti-phospho-p65, and anti-p65 antibodies, and the relative signal density of phosphorylated proteins normalized with that of each total protein in RAW264.7 cells pretreated with U0126 for 2 hours and stimulated with 100 ng/mL LPS for 0.5 hours. Mean of data among three independent experiments. n = 6. Data are shown as means ± S.E. **$p<0.01$, among two groups, as indicated. A.U., arbitrary unit.

Supplementary Figure 3

Full length western blot of Figure 4g

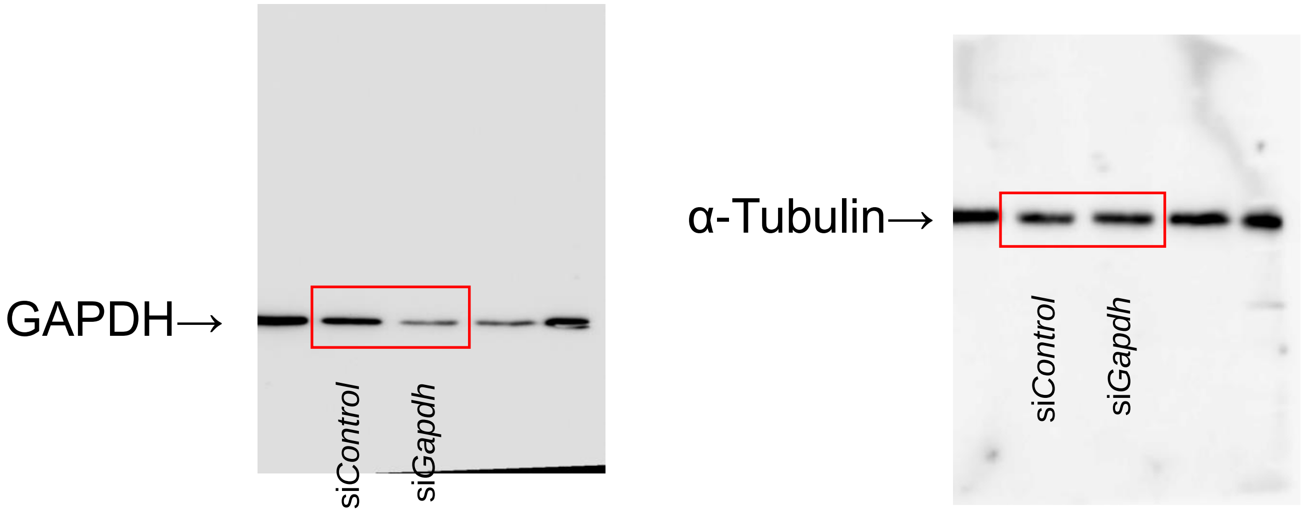

Supplementary Figure 2.
Original full length western blot of Figure 4g. Raw293 cells were transfected with si*Gapdh* or si*Control*. The knockdown efficiency was examined by Immunoblots with anti-GAPDH and anti-α-Tubulin antibodies.

Supplementary Figure 4

a. Full length western blot of Figure 5a

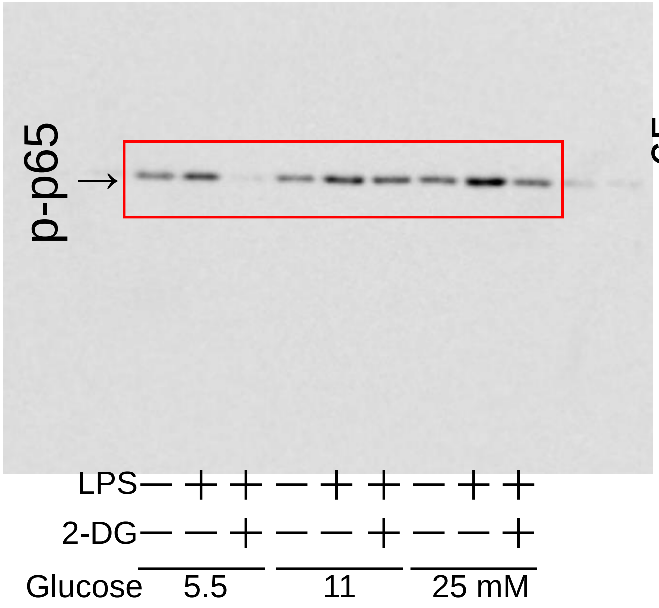

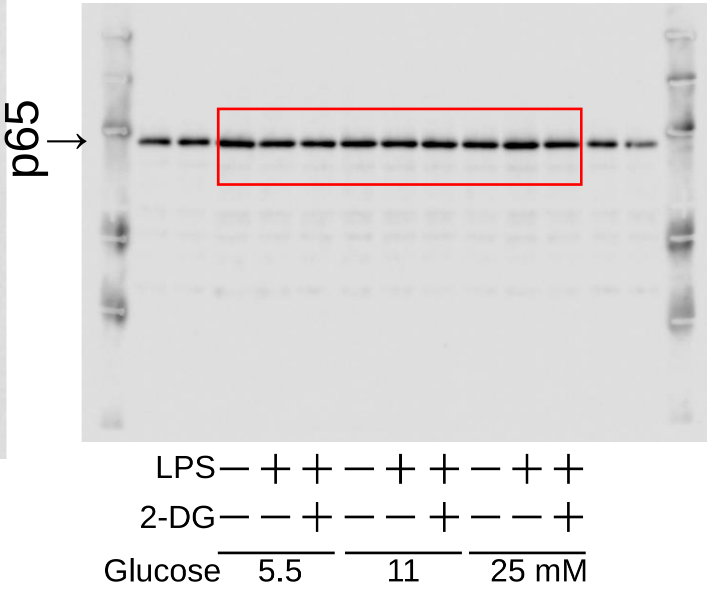

b. Full length western blot of Figure 5b

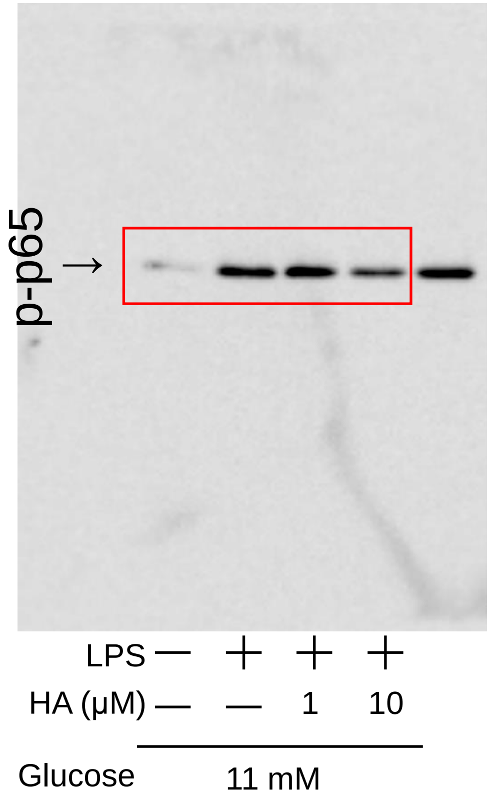

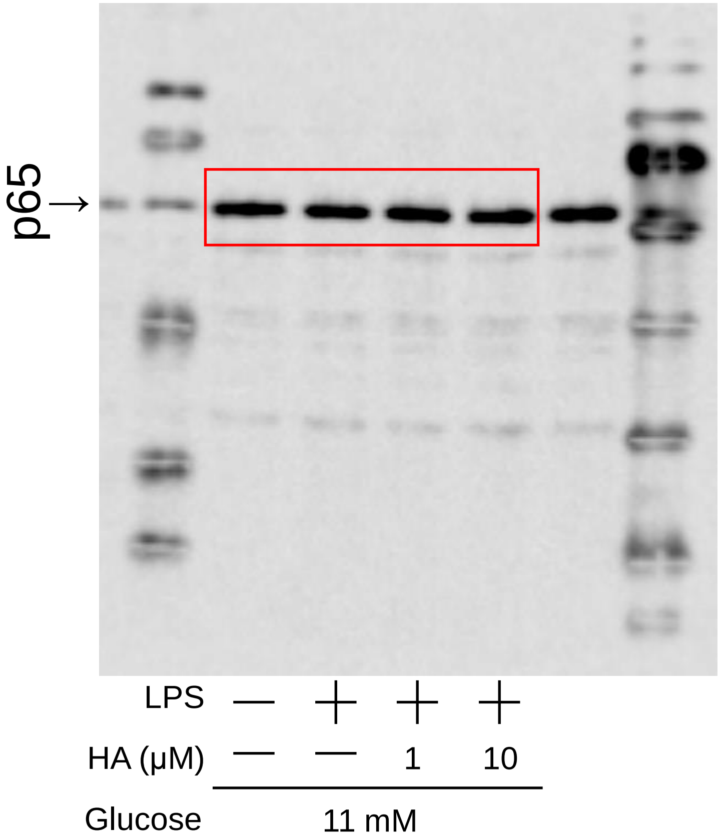

Continue to the next page

Supplementary Figure 4

c. Full length western blot of Figure 5c

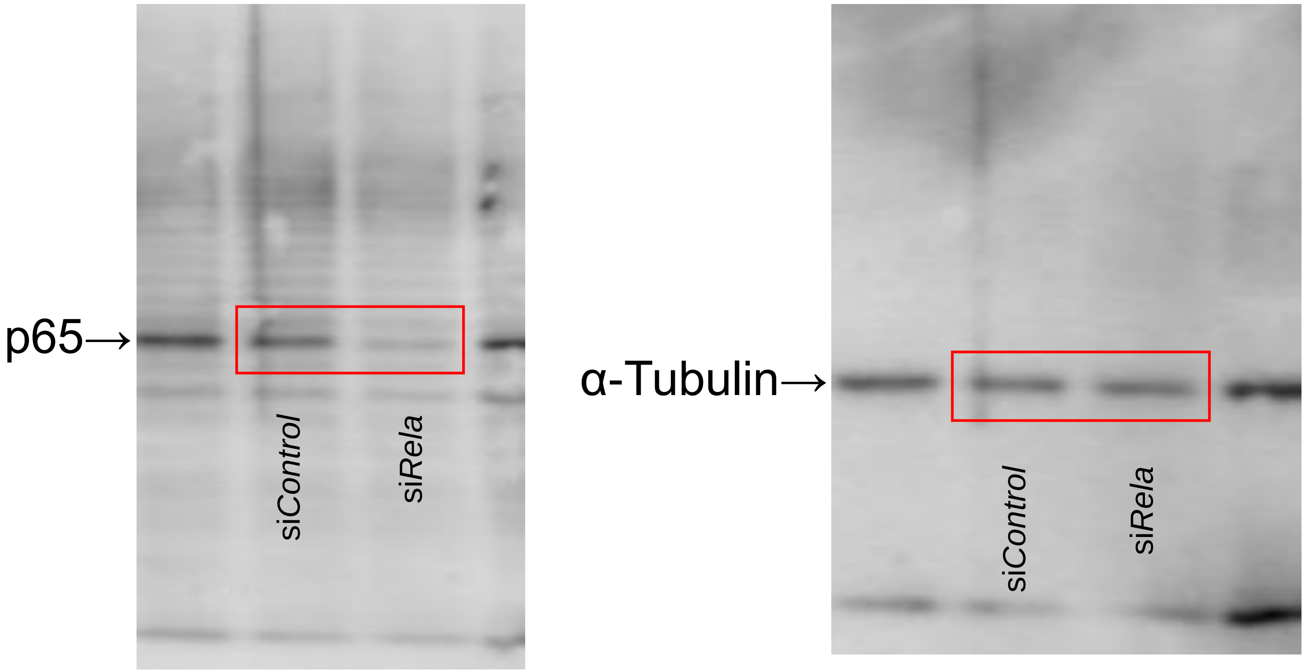

d. Full length western blot of Figure 5f

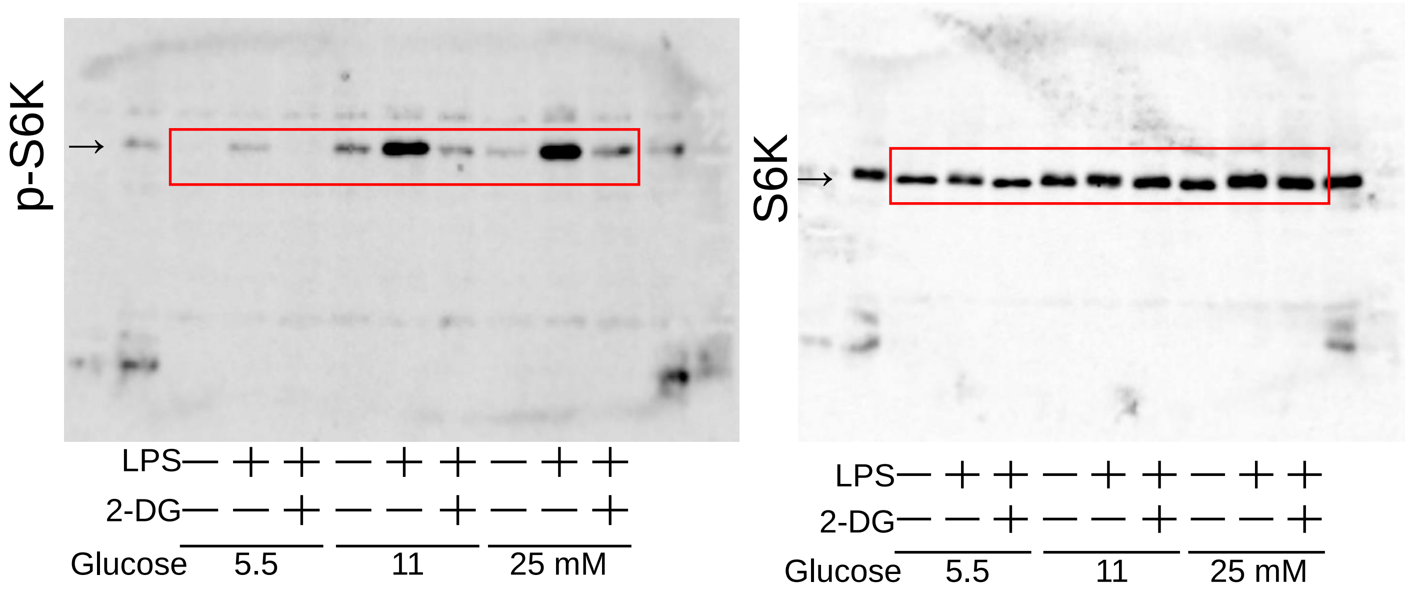

Supplementary Figure 4.
Original full length western blot of Figure 5. (a, b, d) Raw264.3 cells were pretreated with 2DG or HA, and stimulated with LPS for 3h in different glucose concentrations. Harvested cells were subjected to western blotting analysis. (c) Raw264.3 cells were transfected with si*Rela* or si*Control*. The knockdown efficiency was examined by Immunoblots with anti-p65 and anti-α-Tubulin antibodies.

Supplementary Figure 5

a. Full length western blot of Figure 6a

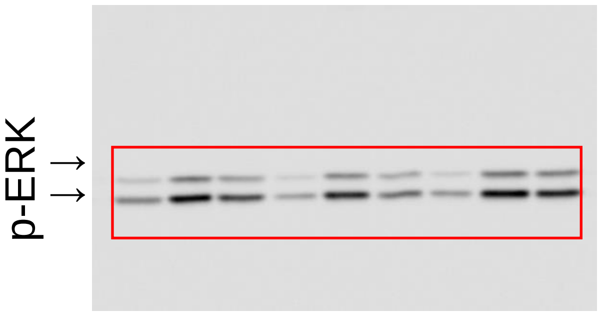

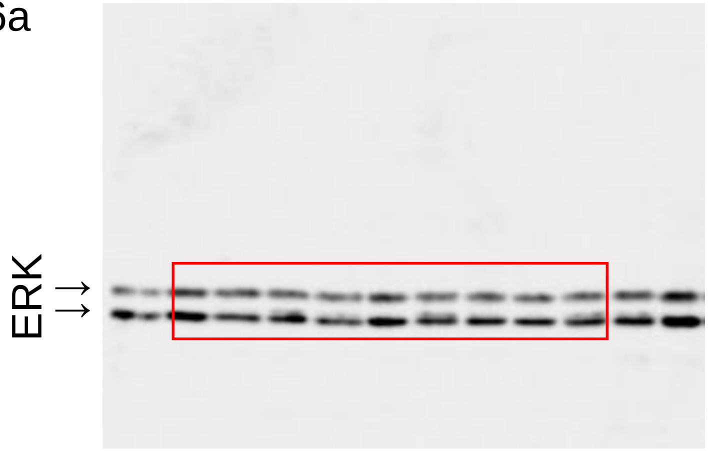

b. Full length western blot of Figure 6b

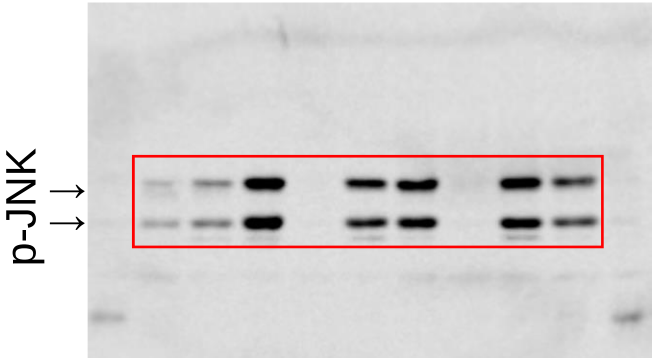

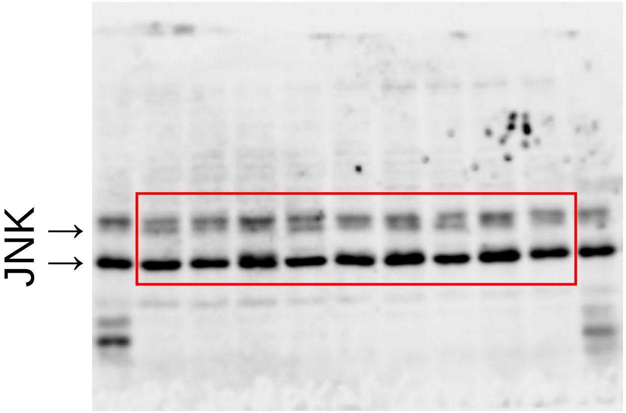

c. Full length western blot of Figure 6c

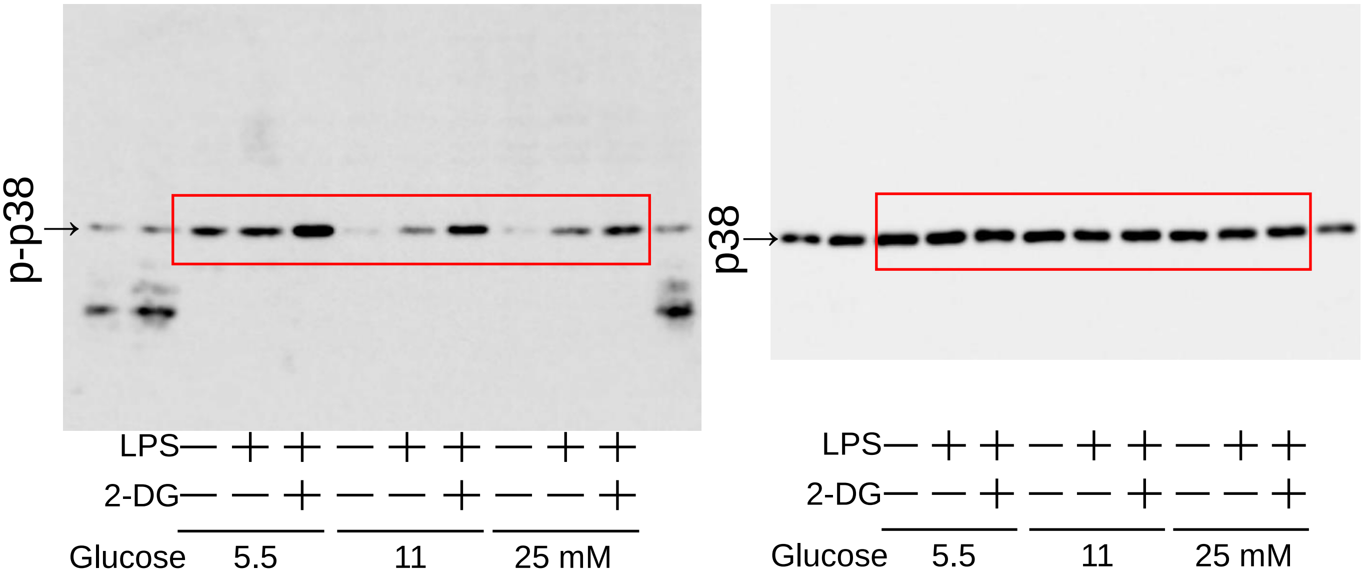

Continue to the next page

Supplementary Figure 5

d. Full length western blot of Figure 6d

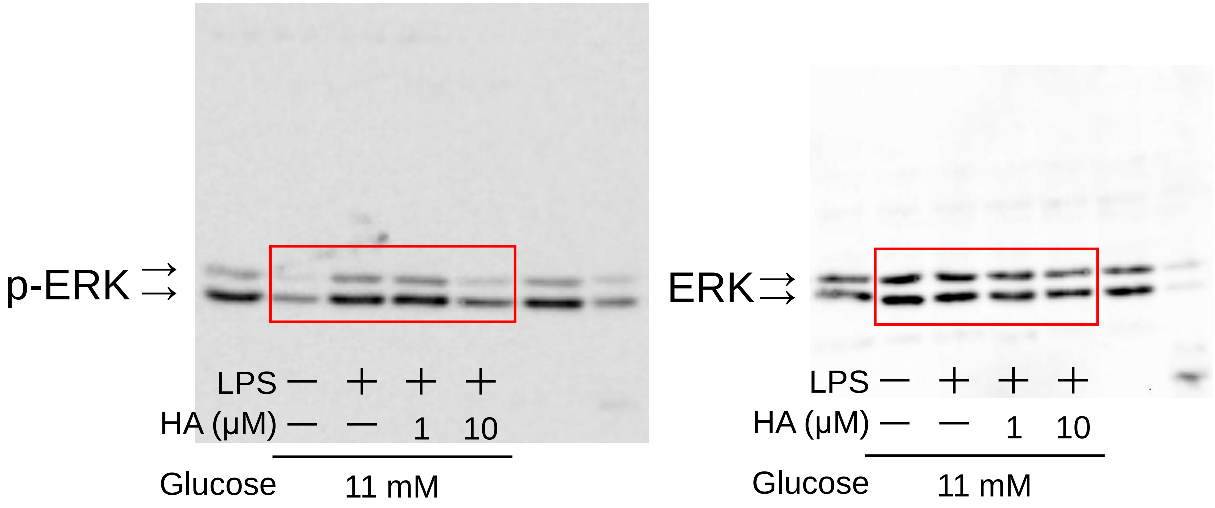

e. Full length western blot of Figure 6e

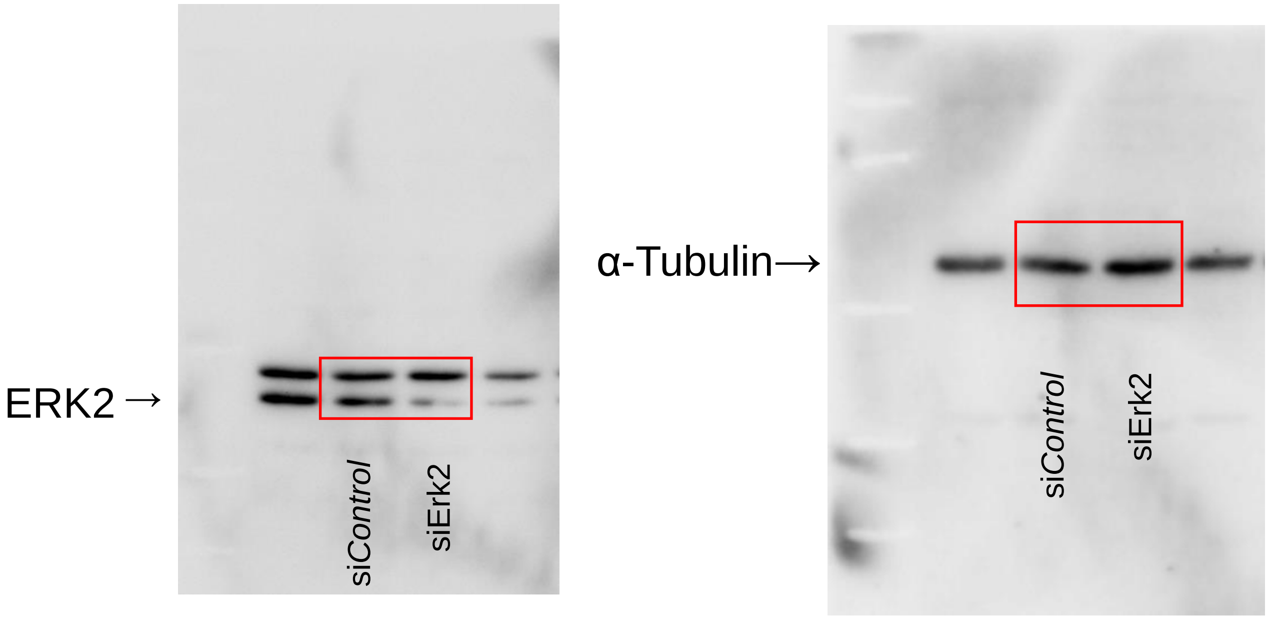

Supplementary Figure 5.
Original full length western blot of Figure 6. (a-d) Raw264.3 cells were pretreated with 2DG or HA, and stimulated with LPS for 3h in different glucose concentrations. Harvested cells were subjected to western blotting analysis. (e) Raw264.3 cells were transfected with si*Erk2* or si*Control*. The knockdown efficiency was examined by Immunoblots with anti-Erk and anti-α-Tubulin antibodies.
